# Review of prostate cancer research in Nigeria

**DOI:** 10.1186/1750-9378-6-S2-S8

**Published:** 2011-09-23

**Authors:** Titilola O Akinremi, Chidiebere N Ogo, Ayodeji O Olutunde

**Affiliations:** 1 Department of Pathology, Federal Medical Centre, Abeokuta, Nigeria; 2 Department of Urology, Federal Medical Centre, Abeokuta, Nigeria

## Abstract

Prostate cancer (CaP) disparities in the black man calls for concerted research efforts. This review explores the trend and focus of CaP research activities in Nigeria, one of the ancestral nations for black men. It seeks to locate the place of the Nigerian research environment in the global progress on CaP disparities. Literature was reviewed mainly through a Pubmed search with the terms “prostate cancer”and “Nigeria”, as well as from internet and hard copies of journal pages.

Findings: One of the earliest publications about CaP in Nigeria was in 1973 from the nation’s 1st tertiary hospital in Ibadan, reporting low incidence, followed by a lull of nearly one decade. In 1980, the incidence rate of CaP was reported as almost similar for black men in Ibadan and Washington and from then on, research work from surgeons and pathologists, from the south to the north, east to west, continued to report increasing prevalence of CaP. Apart from epidemiology, other areas of research include KAP (knowledge attitude and practice) studies (poor education of caregivers and population), histopathology (mostly adenocarcinoma), diagnosis (digital rectal examination [DRE], prostate specific antigen [PSA], ultrasound), clinical features (late presentation and high mortality), and prevention (lifestyle, education and screening). As of today there is a gaping dearth of molecular and genetic studies. Conclusion: The global focus on CaP disparities in black men calls for more efforts from Africa, in all areas of research, along with international collaborations for capacity building.

## Background

Prostate Cancer has become the number one cancer in men with increasing incidence and morbidity in men of black African ancestry [^1^]. Its incidence and prevalence in black men is in multiples of those from other races in several studies [^2^]. The reason for this is not yet clear cut and an explanation for the disparity may lie in studies involving black men from different populations to see if there is an enhancing factor associated with the racial origins of these men. Nigeria is an ancestral home of many black men living outside Africa and it is hoped that an exploration of research activities emanating from the country may shed some light on the disparity [3]. Odedina et al [4] recommend the need to focus on areas of genetic and environmental risk factors in the group.

## Methods

Research studies emanating from Nigeria or involving Nigerian subjects in relation to CaP were sourced using the U.S. National Library of Medicine literature search engine, PubMed [5], using the search terms “prostate cancer” and “Nigeria”. Other sources included journal websites and hard copies of journal articles. These gave reports on CaP from all regions of the country, covering topics including epidemiology, clinical presentation, diagnosis, treatment and prevention.

## Findings

### Epidemiology

In 1973, Nkposong and Lawani[6], urologists from the University College Hospital, Ibadan, South West Nigeria, at the time the only referral center for cancer treatment, noted a low but increasing incidence of prostate cancer. It moved from 8^th^ position of male cancers in 1969 to 2^nd^ place by 1979, with liver cancer leading the pack [7]. In 1981, Udeh [8] also reported the same position from Enugu, but up till 2002, Globocan [9] reported that CaP was not among the top 5 cancers in developing countries.

Angwafo [10], while reporting 93.8/10^5^ incidence from Cameroon in 1994, asked the question “Is prostate cancer rare in black Africa?” while Osegbe [11] in a report from Lagos where the hospital incidence was put at 127/10^5^, surmised that incidence of CaP may be underestimated in Nigerians. Similar reports of increasing hospital based incidence came from other parts of Nigeria with rates of 61.3/10^5^ from Calabar [12] and 182.5/10^5^ from Ife [13]. The stance of Globocan has since changed with the 2008[14] report that CaP had become the top male cancer and fourth commonest cancer in Nigeria.

Ogunbiyi and Shittu [15] from the Ibadan Cancer Registry in 1999 announced a definite increase of CaP among Nigerians. It had risen from 8^th^ position in 1969 to 1^st^ position in 1996, being 11% of all male cancers. Studies from Kano [16], Zaria [17], Benin [18] and Maiduguri [19] showed CaP as16.5%, 9.2%, 7.13% and 6.15% of male cancers.

### Clinical presentation

Reports from all regions of the country emphasize late presentation as the pattern in Nigerian CaP patients. From both South [12] and North [19] about two thirds of patients presented with metastatic disease, and 94.2% [13] and 91% [20] presented with complications respectively. Mortality was generally high with 64% dead within 2 years in a review by Osegbe [11]. Metastases were typically to the spine, with attendant paraparesis or paraplegia, and rare orbital metastases were reported from Ibadan [21].

### Diagnosis

There is growing concern that medical students are not gaining enough skills in DRE [22], such that clinical diagnosis of CaP may become a dilemma for the younger generation of doctors. Though final year students at the University of Jos have adequate teaching, right attitude, perspective, and knowledge about examining for prostate cancer, they have not translated the same into practice and 86% had never felt a malignant prostate while 45% had never examined the gland [23].

Iko [24] in 1987 suggested that prostatic ultrasonography may have great diagnostic promise in developing economies where more sophisticated equipments may be uncommon. However, Ajape [25] in a more recent report(2010) noted 50% sensitivity and false negative correlation between ultrasound and CaP diagnosis.

### Histopathology

Studies from all regions of the country found that adenocarcinoma is the predominant histologic type, similar to global findings. CaP constitutes a moderate portion of biopsies of the prostate. Studies from Benin [18], Ilorin [26] , and Kano [16] also reported malignancy rates of 17%, 16.9% and 22.4% respectively. Freeman et al [27] compared the Gleason system interpretation between genitourinary pathologists in Nigeria, Jamaica and US and concluded that the revealed concordance makes international studies using this grading system feasible.

### PSA

Although (PSA) is a controversial instrument for screening, it remains a useful parameter for monitoring treatment. The distribution of PSA in a rural Nigerian population was found to be similar to that of unscreened US populations with greater than 4ug/l readings in 14% of men [28], and Igwe [29] found that 85.1% prostate cancer patients had total PSA above the normal cut off level. The median total PSA in CaP was found to be 92.6ug/l and 106ug/l respectively in Ibadan [30] and Ife [12].

### Prevention

There is not yet any national cancer screening program and annual PSA checks are not practiced routinely in Nigeria. Lifestyle and behavioral patterns are known to be important in cancer prevention but education about CaP is sparse in Nigeria and opportunistic. Ajape et al [31] concluded that “there is remarkable lack of awareness of prostate cancer among the Nigerian urban populace. Prostate cancer screening and serum PSA test for screening is globally unknown among them”. Though knowledge and risk perception of prostate cancer were low, Oladimeji et al [32] found that 81.5% were willing to be screened for the disease. Odedina et al [33] suggested that emigration of Nigerian men from Nigeria to the US has a significant impact on prostate cancer knowledge and beliefs. Comparing indigenous and immigrant Nigerian men’s diet, alcohol consumption, tobacco use and physical activities, Kumar et al [34] found that there were enough differences to provoke deeper search. Meanwhile, reporting from Nsukka , Ejike and Ezeanyiwa [35] suggested that lifestyle changes in Nigerian men leading to westernized diet and use of energy sparing devices (status symbols) may lead to increase in incidence of chronic diseases like cancer. A trend towards high total plasma omega-6 was observed and found to have a moderate positive correlation with prostate cancer in Nigerians [36].

### Treatment

Since most patients present with poor prognostic features including high histological grades and clinical stages, treatment is mostly palliative with bilateral orchidectomy with or without anti androgen therapy [37]. A discomforting finding in Ilorin showed that only 38.9% of patients had histopathological diagnosis before treatment[25]. No clinical trials were reported.

## Conclusion

Prostate cancer research in Nigeria is growing and multifaceted. There is a need to collate figures into the National Cancer Registry. Education and knowledge about prostate cancer is sparse, and even medical students need better training in digital rectal examination. Patients often present late with complications, pathological diagnosis is mostly of adenocarcinona, resulting commonly in palliative orchiectomy. Routine screening is not practiced and most PSA testing and digital rectal examination emanate from surgical clinics. The disparity in prostate cancer prevalence and mortality in the black man calls for concerted efforts from all and sundry to include all areas of research. Nigerian researchers stand at a vantage position to carry out local work as well as collaborate with other stakeholders all over the world.

## Competing interests

The authors declare that they have no competing interests.
